# Relationship between body size traits and carcass traits with primal cuts yields in Hanwoo steers

**DOI:** 10.5713/ajas.19.0809

**Published:** 2020-06-24

**Authors:** Hyun-Woo Seo, Hoa Van Ba, Pil-Nam Seong, Yun-Seok Kim, Sun-Moon Kang, Kuk-Hwan Seol, Jin-Hyoung Kim, Sung-Sil Moon, Yong-Min Choi, Soohyun Cho

**Affiliations:** 1 National Institute of Animal Science, RDA, Wanju 55365, Korea; 2 Sunjin Meat Research Center, Ansung 17532, Korea; 3 National Institute of Agricultural Sciences, RDA, Wanju 55365, Korea

**Keywords:** Hanwoo, Primal Cuts, Body Size

## Abstract

**Objective:**

This study was conducted to evaluate the correlation between body size traits, carcass traits, and primal cuts in Hanwoo steers.

**Methods:**

Sixty-one beef carcasses were classified for conformation and primal cut weight. Additionally, carcass weight, fat thickness, carcass dimensions, and *longissimus* muscle area were determined to complement the grading.

**Results:**

The average live weight and cold carcass weight were 759 and 469 kg, respectively. The mean carcass meat, fat, and bone proportions were 551, 298, and 151 g/kg, respectively. Primal cuts weights showed significant positive correlations (p<0.001) of 0.42 to 0.82 with live weight, carcass weight, and *longissimus* muscle area and a significant negative correlation with carcass fat (without shank, −0.38 to −0.10). Primal cut weights were positively correlated (p<0.01) with carcass length (0.41 to 0.77), forequarter length (0.33 to 0.57), 6th lumbar vertebrae–heel length (0.33 to 0.59), 7th cervical vertebrae carcass breadth (0.35 to 0.58), 5th to 6th thoracic vertebrae breadth (0.36 to 0.65), 7th to 8th thoracic vertebrae girth (0.38 to 0.63), and coxae girth (0.34 to 0.56) and non-significantly related to cervical vertebrae length and coxae thickness.

**Conclusion:**

There was a high correlation among live weight, carcass weight, *longissimus* muscle area, carcass length, 7th cervical vertebrae carcass breadth, 5th to 6th thoracic vertebrae breadth, and 7th to 8th thoracic vertebrae girth of the primal cuts yield. The correlation between fat and primal cut yields was highly significant and negative. Carcass length and 7th to 8th thoracic vertebrae girth, appear to be the most important traits affecting primal cut yields.

## INTRODUCTION

Beef carcass grading systems are used to estimate the yield and determine the eating quality (palatability) of saleable meat. The meat industry in developed countries is showing trends toward marketing of individual muscle cuts to improve the value of retail meat cuts [1]. Both meat yield and distribution are the primary determinant of carcass value, which affect the value of meat cuts [2]. Studies have been conducted to examine the characteristics affecting carcass yield grade and retail product weight [3–7] and the retail product weight is mainly predicted based on the carcass weight (CW), *longissimus dosi* area, and carcass fat thickness (FT) [8–10]. A few studies have focused on the relationship between carcass scores and carcass composition values. The CW and carcass muscle score account for 38% of total variations in meat yield, with the carcass muscle score accounting for most of this value [11]. The CW alone, CW with carcass muscle score, and CW with carcass muscle and fat scores accounted for 0.1%, 37.9%, and 46.7%, respectively, of the total variation in saleable meat yield [12]. Carcass conformation and fat scores cause moderate to high variation in carcass meat yield [2,12,13]. Recent studies [2,13] showed that carcass classification for conformation and fatness explained 0.55 to 0.70 of total variation in the carcass meat proportion. Studies [12,13] have revealed correlations of 0.6 to 0.7 between muscular scores in the live animal and meat yield. A correlation has been observed between carcass measurements and primal cuts; as the CW increased, the retail product weight increased but the retail cut yield decreased [14]. The objectives of this study were to determine the relationship between instrumental body size traits and carcass traits with primal cuts yields. The fat and bone proportions, derived from body size traits and carcass traits, were used in our predictions.

## MATERIALS AND METHODS

### Animal care

The animal use and protocols employed during the research were reviewed and approved by the Institutional Animal Care and Use Committee (IACUC) at National Institute of Animal Science (approval number NIAS 20001992).

### Animals and management

A total of 61 Hanwoo steers at 28 to 35 months of age was slaughtered at an abattoir of National Institute of Animal Science (Wanju, Korea). The slaughter of the cattle was carried out following the Guidelines of Animal Protection Law (Article 6) [15], and Livestock Sanitation Control Act Law (Annex 1) [16]. After chilling for 21 h at 1°C, the carcasses were weighed and dressing percentage was calculated.

### Carcass evaluations and measurements

**Figure 1:**
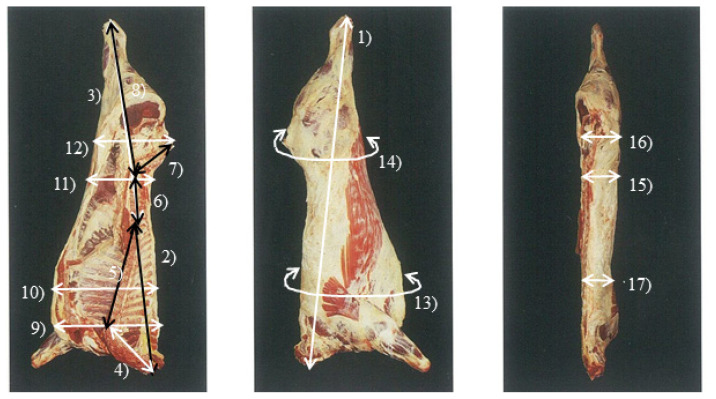
Measurement of carcass size. 1) Carcass length, 2) forequarter length, 3) hindquarter length, 4) cervical vertebrae length, 5) thoracic vertebrae length, 6) lumbar vertebrae length, 7) sacral vertebrae length, 8) 6th lumbar vertebrae to heel length, 9) 7th cervical vertebrae carcass breadth, 10) 5th to 6th thoracic vertebrae breadth, 11) 4th to 5th lumbar vertebrae breadth, 12) 5th sacral vertebrae breadth, 13) 7th to 8th thoracic vertebrae girth, 14) coxae girth, 15) 4th to 5th lumbar vertebrae thick, 16) coxae thick, 17) 7th to 8th thoracic vertebrae thick.

Carcasses were measured for carcass length (CL), forequarter length, hindquarter length, cervical vertebrae length (CVL), thoracic vertebrae length, lumbar vertebrae length, sacral vertebrae length, 6th lumbar vertebrae to heel length, 7th cervical vertebrae carcass breadth (CVB), 5th to 6th thoracic vertebrae breadth (TVB), 4th to 5th lumbar vertebrae breadth, 5th sacral vertebrae breadth (SVB), 7th to 8th thoracic vertebrae girth (TVG), coxae girth, 4th to 5th lumbar vertebrae thick (LVT), coxae thick (CT), 7th to 8th thoracic vertebrae thick (TVT) (Figure 1), FT, and *longissimus* muscle area (LMA). After recording the weight, the carcass was dissected into 10 cuts (tenderloin, loin, strip lion, chuck, clod, top round, bottom round, brisket, shank, and ribs) from which all visible fat and bone (where applicable) was removed. Carcass value was estimated as the sum of the commercial value of each meat cut with a small deduction for bone expressed as a proportion of CW.

### Statistical analysis

Data were analysed using Proc REG and CORR of the Statistical Analysis Systems Institute [17]. Simple correlation coeAfficients of carcass measurements and carcass conformation with the various carcass traits were carried out using Pearson’s correlations.

## RESULTS

### Carcass characteristics

Abbreviations for the measurements collected in the study are provided in Table 1. The means and standard deviations for carcass traits are provided in Table 2. The average live weight (LW) and cold CW were 759.72±59.35 and 469.58± 41.14 kg, respectively. Mean carcass meat, fat, and bone proportions were 551.03±25.89, 297.71±33.70, and 151.27±10.73 kg, respectively. The average primal cuts weight was 8.16±0.89 kg for tenderloin, 38.47±3.74 kg for loin, 10.39±1.09 kg for strip loin, 21.64±2.72 kg for chuck roll, 29.39±3.49 kg for clod, 25.82±2.69 kg for top round, 40.73±4.30 kg for bottom round, 44.47±4.80 kg for brisket, 13.45±2.21 kg for shank, and 58.14 ±6.89 kg for ribs. The carcass characteristics and meat composition data were similar to those found in previous studies [18,19].

### Correlation analyses: carcass traits

Simple correlations between carcass measurements and carcass primal product are presented in Table 3. As expected, the traits most strongly associated with primal cuts weight were LW, CW, and LMA. As CW increased, carcass dimensions and primal cut yields increased [6,20–22]. Based on the values obtained for primal cuts, positive correlations were obtained using the CW with tenderloin proportion (r = 0.78), loin proportion (r = 0.78), strip lion proportion (r = 0.72), chuck roll proportion (r = 0.50), clod proportion (r = 0.72), top round proportion (r = 0.75), bottom round proportion (r = 0.76), brisket proportion (r = 0.76), shank proportion (r = 0.67), and ribs proportion (r = 0.64). Carcass FT showed a positive relationship (r = 0.04 to 0.39) with tenderloin, loin, strip lion, brisket, shank, and ribs but a negative relationship (r = −0.02 to −0.16) with chuck roll, clod, top round, and bottom round. FT was the most useful predictor of percent retail product from the major primal; however, an adjustment concerning the amount of fat in other locations was recommended [23]. Fat cover classification was negatively correlated with the conformation score classification and other muscular development traits such as the leg width and perimeter in both categories [24]. Other studies observed correlations between FT and carcass retail cut ranging from −0.52 to −0.73 [23,25–28].

Positive correlations (p<0.001) were obtained between the LMA and tenderloin proportion (r = 0.54), loin proportion (r = 0.68), strip lion proportion (r = 0.73), chuck roll proportion (r = 0.42), clod proportion (r = 0.50), top round proportion (r = 0.59), bottom round proportion (r = 0.59), brisket proportion (r = 0.52), and ribs proportion (r = 0.55). Correlation coefficients between LMA and retail cut estimates reported previously showed r-values of 0.51 to 0.53 [20,29] and 0.62 to 0.70 [21,25,30,31]. The use of LMA to predict subprimal yield is controversial. Numerous studies have tested the usefulness of LMA for predicting cutability, which revealed a correlation between the ribeye area and both subprimal cut yields and major primal weights (r-values between 0.64 and 0.45) [21,23,32]. However, the USDA yield grade is not strongly influenced by LMA [33–35]. Using separable lean as a dependent variable, the loin eye area showed a low correlation with retail yield [36,37]. LMA exhibited a low but significant correlation with the percentage of retail product for the four primals but was not related to the weight of the retail product [6].

### Correlation analyses: carcass size traits

Correlation coefficients (Table 3) determined using carcass size by CL showed positive correlations with the tenderloin proportion (r = 0.69), loin proportion (r = 0.50), strip lion proportion (r = 0.41), chuck roll proportion (r = 0.22), clod proportion (r = 0.77), top round proportion (r = 0.70), bottom round proportion (r = 0.76), brisket proportion (r = 0.55), shank proportion (r = 0.52), and ribs proportion (r = 0.43). Positive correlations (p<0.001) were obtained between TVG (7th to 8th thoracic vertebrae girth) and the tenderloin proportion (r = 0.59), loin proportion (r = 0.63), strip lion proportion (r = 0.54), clod proportion (r = 0.52), top round proportion (r = 0.61), bottom round proportion (r = 0.61), brisket proportion (r = 0.61), shank proportion (r = 0.42), and ribs proportion (r = 0.62). Negative correlations were obtained for the chuck roll proportion with SVB (r = −0.16), LVT (r = −0.06), CT (r = −0.12), and TVT (r = −0.09). Correlations between the primal cut weight with CVL and CT were poor and generally not significant.

The correlations between variables related to the leg volume, such as carcass width, leg depth, and leg perimeter, were highly significant for both commercial categories. However, the correlations among these muscle-related variables and those related to carcass size or skeletal size, such as CL and width between commercial categories [24]. The CL was positively related to the percentage of subprimal cuts from round and brisket, chuck, plate, and flank [38].

## CONCLUSION

Development of a new prediction equation for determining Hanwoo yield is required because of changes in the Korean beef industry; for example, the proportion of steers has dramatically increased, with slaughter weight of up to 760 kg. Our results showed that numerous variables in combination with body size or carcass traits can be used to more accurately estimate fat and carcass yields. There was a high correlation among LW, CW, LMA, CL, CVB, TVB, and TVG and primal cut yield. The correlation between fat and primal cut yields was highly negatively significant. The CL and TVG showed a high correlation with carcass yield. The relationship between carcass conformation and body size with carcass traits provides a basis for developing a carcass pricing structure that better reflects carcass value in terms of meat yield and distribution.

## Figures and Tables

**Table 1:** Description of acronyms

Acronym	Definition
LW	Live weight
CW	Cold carcass weight
DP	Dressing percentage (cold carcass weight×100/live weight)
LMA	*Longissimus dosi* area
FT	Carcass fat thickness
CL	Carcass length
FL	Forequarter length
HL	Hindquarter length
CVL	Cervical vertebrae length
TVL	Thoracic vertebrae length
LVL	Lumbar vertebrae length
SVL	Sacral vertebrae length
LVHL	6th Lumbar vertebrae – heel length
CVB	7th Cervical vertebrae carcass breadth
TVB	5th to 6th Thoracic vertebrae breadth
LVB	4th to 5th Lumbar vertebrae breadth
SVB	5th Sacral vertebrae breadth
TVG	7th to 8th Thoracic vertebrae girth
CG	Coxae girth
LVT	4th to 5th Lumbar vertebrae thick
CT	Coxae thick
TVT	7th to 8th Thoracic vertebrae thick

**Table 2:** Mean, standard deviation (SD), minimum and maximum values of carcass traits and primal cuts yields1)

Items	Mean	SD	Minimum	Maximum
LW (kg)	759.72	59.35	627.00	900.00
CW (kg)	469.58	41.14	393.70	567.50
FT (mm)	16.33	6.71	7.00	40.00
LMA (cm^2^)	94.48	8.74	75.00	112.00
DP (%)	61.79	1.77	58.40	66.18
Meat (g/kg)	551.03	25.89	500.50	613.87
Fat (g/kg)	297.71	33.70	229.08	363.15
Bone (g/kg)	151.27	10.73	130.46	174.66
CL (cm)	264.46	8.57	244.00	285.00
FL (cm)	114.79	4.84	101.00	124.00
HL (cm)	150.28	5.55	139.00	163.00
CVL (cm)	45.89	5.54	38.00	84.00
TVL (cm)	80.08	3.76	65.00	86.00
LVL (cm)	42.37	2.55	35.00	50.00
SVL (cm)	35.52	3.48	27.00	42.00
LVHL (cm)	107.54	7.24	84.00	130.00
CVB (cm)	81.62	3.57	70.00	89.00
TVB (cm)	81.70	3.20	75.00	90.00
LVB (cm)	45.26	2.95	39.00	53.00
SVB (cm)	50.48	3.45	45.00	62.00
TVG (cm)	179.54	5.46	168.00	188.00
CG (cm)	132.85	4.88	122.00	144.00
LVT (cm)	25.13	3.50	18.00	34.00
CT (cm)	21.69	2.83	14.00	28.00
TVT (cm)	21.00	3.17	13.00	30.00
Tenderloin (kg)	8.16	0.89	6.61	10.39
Loin (kg)	38.47	3.74	29.50	46.86
Strip loin (kg)	10.39	1.09	8.37	13.31
Chuck roll (kg)	21.64	2.72	15.67	27.94
Clod (kg)	29.39	3.49	23.43	39.25
Top round (kg)	25.82	2.69	20.35	32.20
Bottom round (kg)	40.73	4.30	32.53	48.67
Brisket (kg)	44.47	4.80	34.10	57.75
Shank (kg)	13.45	2.21	8.15	19.57
Ribs (kg)	58.14	6.89	42.53	75.02

LW, live weight; CW, cold carcass weight; FT, carcass fat thickness; LMA, *Longissimus dosi* area; DP, dressing percentage (cold carcass weight×100/live weight); CL, carcass length; FL, forequarter length; HL, hindquarter length; CVL, cervical vertebrae length; TVL, thoracic vertebrae length; LVL, lumbar vertebrae length; SVL, sacral vertebrae length; LVHL, 6th lumbar vertebrae – heel length; CVB, 7th cervical vertebrae carcass breadth; TVG, 7th to 8th thoracic vertebrae girth; CG, coxae thick; LVT, coxae girth, 4th to 5th lumbar vertebrae thick; CT, coxae thick; TVT, 7th to 8th thoracic vertebrae thick.

1) Beef primal cuts [39].

**Table 3:** Simple correlation coefficients between carcass grades, carcass weight, fat thickness, carcass size and yield of primal product

Trait	Beef primal cuts1)									

	Tenderloin	Loin	Strip loin	Chuck roll	Clod	Top round	Bottom round	Brisket	Shank	Ribs
LW (kg)	0.81***	0.76***	0.65***	0.46***	0.77***	0.81***	0.82***	0.75***	0.61***	0.65***
CW (kg)	0.78***	0.78***	0.72***	0.50***	0.72***	0.75***	0.76***	0.76***	0.67***	0.64***
FT (mm)	0.04	0.04	0.15	−0.16	−0.02	−0.07	−0.04	0.16	0.39**	0.05
LMA (cm^2^)	0.54***	0.68***	0.73***	0.42***	0.50***	0.59***	0.59***	0.52***	0.24	0.55***
DP (%)	0.16	0.30*	0.40**	0.28*	0.10	0.06	0.09	0.23	0.38**	0.16
Meat (g/kg)	0.31*	0.21	0.15	0.14	0.38**	0.39**	0.37**	0.21	−0.26*	0.06
Fat (g/kg)	−0.28*	−0.16	−0.08	−0.10	−0.38**	−0.38**	−0.38**	−0.13	0.31*	−0.06
Bone (g/kg)	0.13	0.01	−0.10	−0.03	0.28*	0.26*	0.29*	−0.09	−0.35**	0.06
CL (cm)	0.69***	0.50***	0.41***	0.22	0.77***	0.70***	0.76***	0.55***	0.52***	0.43***
FL (cm)	0.56***	0.47***	0.33**	0.39**	0.57***	0.47***	0.56***	0.41**	0.41**	0.33*
HL (cm)	0.50***	0.31*	0.20	0.01	0.56***	0.50***	0.53***	0.38**	0.35**	0.30*
CVL (cm)	0.23	0.24	0.13	0.20	0.17	0.24	0.23	0.15	0.23	0.14
TVL (cm)	0.42***	0.32**	0.20	0.31*	0.34**	0.36**	0.41**	0.15	0.29*	0.23
LVL (cm)	0.29*	0.24	0.08	0.05	0.27*	0.28*	0.21	0.18	0.22	0.12
SVL (cm)	0.28*	0.19	0.34**	0.06	0.30*	0.32*	0.29*	0.40*	−0.04	0.32*
LVHL (cm)	0.57***	0.55***	0.41**	0.29*	0.59***	0.57***	0.58***	0.58***	0.33*	0.48***
CVB (cm)	0.51***	0.45***	0.43***	0.35**	0.52***	0.55***	0.58***	0.52***	0.44***	0.39**
TVB (cm)	0.50***	0.47***	0.49***	0.41***	0.45***	0.64***	0.65***	0.38**	0.41***	0.36**
LVB (cm)	0.43***	0.29*	0.46***	0.09	0.40**	0.37**	0.50***	0.35**	0.33*	0.21
SVB (cm)	0.35**	0.32*	0.32*	−0.16	0.35**	0.26*	0.29*	0.38**	0.22	0.32*
TVG (cm)	0.59***	0.63***	0.54***	0.38**	0.52***	0.61***	0.61***	0.61***	0.42***	0.62***
CG (cm)	0.44***	0.39**	0.56***	0.52***	0.39**	0.49***	0.53***	0.40**	0.34**	0.35**
LVT (cm)	0.31*	0.24	0.34**	−0.06	0.39**	0.37**	0.38**	0.39**	0.42***	0.11
CT (cm)	0.05	0.08	0.18	−0.12	0.10	0.09	0.11	0.18	0.20	−0.02
TVT (cm)	0.26*	0.10	0.14	−0.09	0.35**	0.22	0.21*	0.27*	0.22	0.04

LW, live weight; CW, cold carcass weight; FT, carcass fat thickness; LMA, *longissimus dosi* area; DP, dressing percentage (cold carcass weight×100/live weight); CL, carcass length; FL, forequarter length; HL, hindquarter length; CVL, cervical vertebrae length; TVL, thoracic vertebrae length; LVL, lumbar vertebrae length; SVL, sacral vertebrae length; LVHL, 6th lumbar vertebrae – heel length; CVB, 7th cervical vertebrae carcass breadth; TVB, 5th to 6th thoracic vertebrae breadth; LVB, 4th to 5th lumbar vertebrae breadth; SVB, 5th Sacral vertebrae breadth; TVG, 7th to 8th thoracic vertebrae girth; CG, coxae thick; LVT, coxae girth, 4th to 5th lumbar vertebrae thick; CT, coxae thick; TVT, 7th to 8th thoracic vertebrae thick.

1) Beef primal cuts [39].

* p<0.05,

** p<0.01,

*** p<0.001.
